# Feasibility of a Single-Session Electrodermal Biofeedback Intervention for State Anxiety

**DOI:** 10.1007/s10484-025-09720-2

**Published:** 2025-06-12

**Authors:** Peter Dobo, Krisztian Kasos

**Affiliations:** Institute of Psychology, ELTE Eötvös Loránd University, Izabella U. 46, Budapest, 1064 Hungary

**Keywords:** Alternative feedback sites, Biofeedback, Electrodermal, State anxiety

## Abstract

Anxiety—a prevalent mental health issue—is on the rise, leading to physical health problems, burnout, and societal challenges. Technological advances and limited mental health care have driven individuals toward self-monitoring devices, with biofeedback emerging as a key method for emotional regulation. Electrodermal biofeedback—though widely used—has shown mixed results in stress and anxiety management. Moreover, measurement sites for self-monitoring devices tend to be non-traditional sites such as the wrist. This study aims to assess the feasibility of a one-time electrodermal biofeedback session on state anxiety and evaluate the wrist as a viable feedback site. A randomized controlled primary study (*N* = 110) and a follow-up study (*N*  = 39) to confirm the results of the first study were conducted. Participants were randomized into control, feedback from fingers and feedback from the wrists conditions followed by a 10-min biofeedback session. Our results confirm the efficacy of a 10-min biofeedback session on self-reported state anxiety and skin conductance level, response amplitude and the number of non-specific responses. We found no significant differences between feedback received from the wrists, and feedback from the fingers. Additionally, our findings suggest that skin conductance level, response amplitude and the number of non-specific responses do not show a clear relationship with self-reported anxiety. In conclusion, there is evidence of feasibility of electrodermal biofeedback in managing state anxiety, and the wrist shows promise to be a viable site for biofeedback in anxiety management. Future research should explore the interactions between electrodermal activity and self-reported measures of anxiety to optimize biofeedback interventions.

## Introduction

Stress—a complex reaction triggered by environmental, social, or psychological stressors—induces a spectrum of temporary biological, behavioral, and cognitive changes aimed at enhancing adaptive behavior (Hellhammer et al., 2010). This physiological and psychological phenomenon, particularly when prolonged, is intricately linked to the deterioration of both physical and mental health, manifesting in issues like burnout, depression, anxiety and cardiovascular diseases. Anxiety—one of the most common stress related mental health problems—is characterized by hyper-vigilance to threat, which also includes selective attention towards and difficulty disengaging from threat related information (Attwood et al., 2017; Leleu et al., 2014). The escalating prevalence of anxiety disorders, potentially impacting up to one-third of the population over their lifetime (Bandelow & Michaelis, 2015), is concerning as well as straining the capacity of mental health services (Morina et al., 2012). The economic burden of anxiety disorders is also significant, costing, for example, the European Union billions of euros annually due to healthcare expenses, productivity losses, and premature labor force exit (Gustavsson et al., 2011).

To define the construct of anxiety authors were led by its different aspects. Lewis (1970) highlights anxiety as an emotional state that is closely related to perceived fear. According to Spielberger (1966) two kind of anxiety can be distinguished based on the specificity or generality of the situation. State anxiety is the emotional state experienced in a specific situation that changes relatively quickly, therefore, momentarily state anxiety and physiological arousal are corresponding. Meanwhile trait anxiety is more like a personality trait, regarding how much one is affected, it is stable in time and universal in different situations. This concept is supported by genetic research that found moderate correlation between genetic factors and the levels of anxiety (Lau et al., 2006).

Cognitive Behavioral Therapy (CBT)—particularly exposure therapy—represents the gold standard for treating anxiety disorders (Craske et al., 2014). Despite its established efficacy (Craske et al., 2018), a notable fraction of patients fails to respond to it or encounters a recurrence of fear and anxiety post-treatment (Craske et al., 2014). CBT and psychopharmacology often overlook the physiological dysregulation inherent in anxiety (Bandelow & Michaelis, 2015; Bandelow et al., 2017). Specifically, CBT predominantly addresses the cognitive dimensions of anxiety, neglecting the concomitant sympathetic arousal (Weerdmeester et al., 2020). Recent discourse in the field has advocated for incorporating cognitive-emotional processes into CBT, particularly focusing on emotion regulation through amygdala inhibition and the enhancement of self-efficacy (Craske et al., 2018)- elements closely aligned with the mastery of biofeedback techniques (Weerdmeester et al., 2020). Thus, examining the benefits and deciphering the mechanism behind biofeedback becomes increasingly important. Furthermore, investigating whether an intervention effectively interacts with its intended physiological or psychological mechanism and whether modifications in this mechanism lead to measurable improvements in outcomes is ever more critical. Based on these investigations existing interventions may be improved and novel interventions designed in the future.

Biofeedback allows one to gain control over automatic processes by measuring and feeding back information about the peripheral nervous system to aid the acquisition of conscious control over them (Pop-Jordanova & Pop-Jordanov, 2002). Biofeedback has a long history in the treatment of different physical and mental disorders, and it shows some evidence of helping people combat stress, both chronic and acute (Tinello et al., 2022). Different biofeedback methods exist, such as electrodermal biofeedback, which targets directly the sympathetic nervous system. Electrodermal biofeedback involves monitoring changes in eccrine sweat gland activity, a reflection of the sympathetic nervous system's response to stress (Boucsein, 2012; Dawson et al., 2007). Skin conductance measurement—a method for electrodermal activity (EDA) assessment—encompasses phasic and tonic components, each reflecting sympathetic nervous system (SNS) activity. The phasic component or skin conductance response (SCR) quantifies rapid fluctuations, primarily associated with cognitive and emotional processing. The tonic component, or skin conductance level (SCL), represents SNS baseline activity, serving as an indicator of overall arousal (Dawson et al., 2007).

Over the years, the research on the efficacy of electrodermal biofeedback in managing stress and anxiety has yielded mixed results, with only a handful of studies contributing to the field. Some research has explored EDA biofeedback as a complementary treatment alongside other stress management techniques, making it challenging to isolate the effects of biofeedback alone (Bhambri, 2021; Pop-Jordanova & Pop-Jordanov, 2002; Raaijmakers et al., 2013; Rusciano et al., 2017; Yahav & Cohen, 2008). Additionally, some studies lacked control groups, which complicates the assessment of its efficacy. Results from these studies have ranged from reductions in anxiety (Khanna et al., 2007; Palekar et al., 2015) to no impact or even increases in self-reported stress (MacLean et al., 2013). When control conditions are used, outcomes vary significantly. Some studies report minor benefits (Teufel et al., 2013), while others note substantial effects (Biswas et al., 1995; Dillon et al., 2016; Janka et al., 2017). Other research has found positive effects on cognitive test performance, an alternative method of measuring stress and anxiety reduction (Parnandi, 2017). However, contrasting these findings, a triple-blind randomized controlled trial (RCT) reported no significant effects of EDA biofeedback on physiological measures, however this study did not directly assess self-report anxiety (Raaijmakers et al., 2013). Given these varied and sometimes contradictory results, it remains challenging to definitively conclude the overall efficacy of EDA biofeedback in reducing anxiety. Additionally, the lack of published EDA data in the cited articles is notable. Out of the studies referenced previously, only 4 included EDA data, collected during biofeedback, and did that to only a limited extent, certainly without detailed scrutiny (Parnandi & Gutierrez-Osuna, 2017; Raaijmakers et al., 2013; Rusciano et al., 2017; Yahav & Cohen, 2008). This is unexpected, considering that all these studies incorporated EDA biofeedback in their design; one might anticipate a more detailed analysis of the underlying mechanism. Consequently, our understanding of EDA dynamics during biofeedback sessions remains limited.

The prevalence and increasing popularity of self-monitoring by smart devices that measure a wide range of physiological indicators, in many cases, measure EDA from non-traditional measurement sites such as the wrist. Since non-traditional measurement sites have lower correlation with traditional measurement sites (Kasos et al., 2020b), it is not at all evident that feeding information back from these sites will be similarly effective as traditional measurement sites (the nondominant palmar surfaces) in reducing physiological arousal or anxiety. This raises questions about the potential efficacy of providing biofeedback from different body sites in stress and anxiety management.

Adding further complexity to the field, studies have shown mixed results regarding the relationship between self-reported anxiety and autonomic responses. Some found no difference in electrodermal activity between individuals with high and low trait anxiety, suggesting that EDA may not always correspond to self-reported anxiety levels (Kilpatrick et al., 2006). Similarly, others found no correlation between self-reported anxiety and EDA in individuals with a drug dependency, further highlighting the complexity of this relationship (Scheutz, 1986). Strohmaier and colleagues (2020) found a significant interaction effect between trait anxiety and EDA. Furthermore, Rosebrock et al. (2017) noted that individuals with anxiety disorders might exhibit heightened self-reported arousal without corresponding increases in skin conductance reactivity, suggesting the relationship is not always straightforward. Moreover, some found that highly anxious individuals had lower EDA, pointing to a potential discrepancy between self-reported and physiological indicators of anxiety (Naveteur & Freixa I Baque, 1987). Importantly, in an experiment, in which participants were trained to elevate their EDA, researchers found no change in anxiety between pre- and post-measures (Schoenberg et al., 2012). This also calls in question the notion that EDA feedback has a linear relationship with self-reported anxiety. These findings underline the need to further assess the relationship between EDA and self-reported anxiety, especially when EDA is used as a biofeedback measure for anxiety management.

The purpose of the present study is to evaluate the efficacy of a one-time electrodermal biofeedback session in self-reported anxiety and on SCL, SCR and non-specific responses. The study also aims to evaluate the efficacy of the wrist as a potential electrodermal feedback site. Moreover, we aim to elucidate the relationship between self-report anxiety and SCL, SCR and non-specific responses. In order to accomplish these goals, we conducted a primary and a follow up randomized controlled study.

## Study 1

### Methods

#### Sample

The study was approved by the institutional ethical board of Eötvös Loránd University (ELTE) and recruited a total of 113 participants (84 females and 29 males). Participants were recruited through a research call directed at students enrolled in a course specifically designed to involve participation in research studies. The call explained that the study aimed to investigate the cognitive and physiological components of biofeedback’s anxiety-reducing effects. Interested students were informed that participation would involve completing questionnaires and taking part in a laboratory experiment. The average age of participants was 21.5 years (SD = 3.1), all of whom were students from various faculties of the university and took part in the study for course credit. The study was preregistered on Open Science Framework (10.17605/OSF.IO/4M7PV).

#### Procedure

**Fig. 1:**
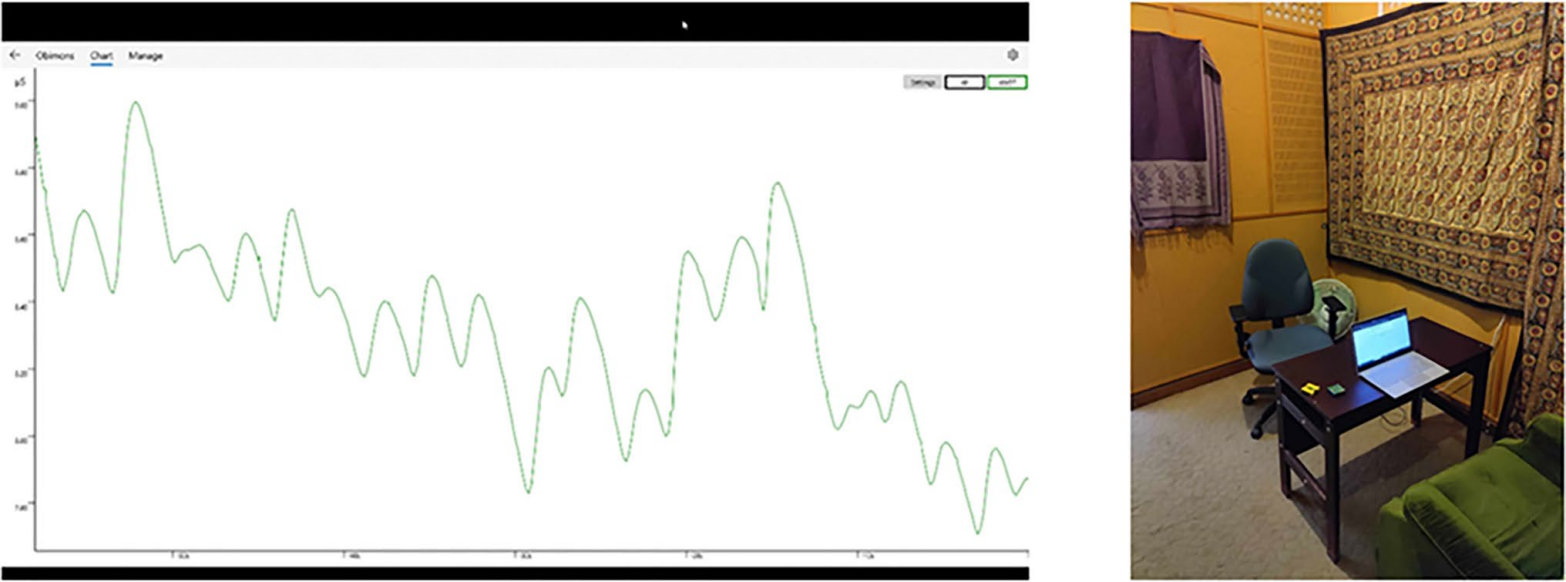
Biofeedback and experimental setup. *Note.* Screen records are also available for some of the participants and individual graphs of the sessions on this link: https://osf.io/7kjf9/?view_only=ee5275e19ad3498a83aa8de8c28728f0

Participants first filled out informed consent which was followed by a battery of questionnaires the evening before the experiment, which, aside from demographic data (gender, age), included questionnaires detailed in the instruments section (only questionnaires related to stress and anxiety will be discussed here). The experiment was conducted in a sound attenuated room with a constant temperature of approximately 24 °C (Fig. 1).

Participants were randomly assigned to 5 different conditions (control and experimental conditions differentiated by biofeedback from fingers on the dominant hand, fingers on the nondominant hand, dominant wrist, or nondominant wrist). Upon arrival, participants first completed the STAI state anxiety questionnaire. Following the completion of the questionnaire, devices were placed according to condition: If biofeedback was provided from the fingers, devices were placed on the distal phalanges of the middle and index fingers on both hands. If biofeedback was provided from the wrists, biofeedback devices were placed approximately 2–3 cm proximally from the distal end of the forearm on the ventral side of either the left or right arm (depending on condition), and the reference device was placed on the distal phalanges of the middle and index fingers of the nondominant hand. For each participant, the nondominant hand’s index and middle fingertips served as reference measurement site, except receiving biofeedback from the nondominant fingers, in this case, the reference measurement point was the fingers of the dominant hand. The nondominant hand was chosen as a reference site to enable comparison with many previous studies used this site to provide biofeedback from (Nagai et al., 2004; Pop-Jordanova & Pop-Jordanov, 2020; Schoenberg et al., 2012; Steinberg & Schwartz, 1976). In the control condition, EDA data were collected from the nondominant hand's fingers and wrist.

Once devices were attached, participants received instructions that included information on EDA and the presumed process behind the stress-reducing effect of biofeedback. They were advised on how to decrease their skin conductance, which they could monitor via a curve displayed on a screen in front of them (Fig. 1). This was followed by a 10-min biofeedback session (during which the experimenter left the room). After the session, the devices were removed by the experimenter, and participants once again completed the STAI state anxiety questionnaire. The control group differed in that, instead of a biofeedback intervention, they read a piece of classical literature (Hamlet, Prince of Denmark) for 10 min. The instructions provided were identical for both control and experimental groups, except for the omission of methods to decrease EDA for the control group. Both experimental groups and the control group were aware that the purpose of the study was anxiety reduction.

At the end of the study, all participants had the opportunity to ask questions about the research, and members of the control group were offered the chance to participate in a biofeedback session identical to that of the experimental group.

#### Instruments

- The State-Trait Anxiety Inventory (STAI) is a frequently used 20-item measure for assessing anxiety. Participants rate each item on a scale from 1 (Not at all) to 4 (Very much so), depending on how much they feel the statement applies to them at that moment (STAI-S) or in general (STAI-T) (Spielberger et al., 1970).
- The Depression Anxiety Stress Scales-21 (DASS-21) measures three related negative emotional states: depression, anxiety, and stress/tension. Responses are rated from 0 (Did not apply to me at all) to 3 (Applied to me very much) based on the past week (Henry & Crawford, 2005).
- The Hungarian adaptation of the Perceived Stress Scale (PSS) assesses the degree of stress perceived by an individual over the past month, with responses rated on a 5-point Likert scale (Cohen et al., 1983).

#### Equipment and Data Processing

To measure skin conductance, the Open-Source Biomonitor (Obimon) was used, which operates at a sampling frequency of 8 Hz (Kasos et al., 2019). Skin conductance data were captured using disposable Ag/AgCl electrodes (dimensions: 32 × 41 mm, Skintact FS-RG1; Leonhard Lang GmbH, Innsbruck, Austria), with electroconductive gel pre-applied to each electrode to ensure optimal conductivity between the electrode and the skin's surface.

The integrity of the raw data was evaluated for any artifacts through visual inspection. Data segments identified to contain artifacts were subsequently excluded from further analysis. Data were also lost because of faulty equipment or electrodes losing contact. Altogether 77 participants’ data (both from feedback and reference site) remained in the analysis.

EDA data were processed using Ledalab version 3.4.8 (Benedek & Kaernbach, 2010a, b). Initial data processing involved Gaussian smoothing to mitigate error noise, followed by the derivation of SCL and SCR through the application of optimized Continuous Decomposition Analysis. Analysis windows of 30 s were systematically employed to extract metrics such as average SCL, non-specific responses, and response amplitudes. For the identification of non-specific SCRs, a minimum response threshold of 0.01 microSiemens was established.

#### Biofeedback

Feedback was provided via a laptop using Obimon’s native application. The laptop was placed on a desk approximately 60 cm away from the participants ensuring that participants were seated in a comfortable position. EDA data was presented as a real-time line chart for the participants (See Fig. 1 for details).

#### Statistical Analysis

##### Self-Report State Anxiety

Repeated measure ANOVA was used with the within subject effect of Time (pre and post intervention) and the between subject effect of Condition (Control and Experimental conditions).

##### Skin Conductance Level, Non-specific Response Amplitudes and the Number of Non-specific Responses

Repeated measure ANOVA was used with the within subject effect of Time (first 30 s and last 30 s of intervention, referred to as Pre and Post intervention) and Measurement site (Feedback site and Reference site), and the between subject effect of Group (Control and Experimental groups).

##### Correlation Between Self-report Anxiety and Physiological Measures

STAI-state pre and post session scores were correlated with measured SCL, number of responses and response amplitudes. Change in STAI scores were calculated by taking the difference between post and pre scores (the smaller the number the bigger the reduction in self-report anxiety). Change in SCL was calculated by taking the difference between the average of the last 30 s and the average of the first 30 s SCL. The smaller the value the bigger the reduction in SCL was observed. The same procedure was conducted with the number of non-specific responses and the response amplitudes. These calculated scores were also correlated with STAI-state and change scores.

## Results

One-way ANOVA was conducted to reveal any differences in trait anxiety, stress, and depression among experimental groups. The analysis resulted in a significant difference between conditions on the dependent variable DASS-depression *F* (4,105) = 2.98, *p* = 0.023, η_p_^2^ = 0.10. Participants in the condition receiving biofeedback from the nondominant fingers reported significantly lower depression scores compared to the condition receiving biofeedback from the dominant wrist. There were no other significant differences (See Table 1 for details).

**Table 1:** Descriptive statistics of the Battery of questionnaire

Biofeedback Groups					
Self-report measures	Control group *N* = 18	Nondominant fingers *N* = 23	Dominant fingers *N*= 24	Dominant wrist *N* = 23	Nondominant wrist *N* = 22
Stai Trait	46.39 (± 6.51)	44.39 (± 7.61)	48.04 (± 6.29)	48.00 (± 9.43)	42.05 (± 9.21)
Dass Stress	9.83 (± 3.40)	8.39 (± 4.37)	9.92 (± 4.38)	9.52 (± 4.91)	7.05 (± 3.17)
Dass Anxiety	6.11 (± 4.03)	4.04 (± 3.52)	5.96 (± 5.64)	5.30 (± 5.10)	3.32 (± 3.54)
Dass Depression	5.89 (± 3.34)	3.87 (± 3.14)	6.21 (± 4.41)	7.61 (± 4.87)	4.64 (± 3.90)
Perceived Stress Scale	31.56 (± 6.52)	28.09 (± 6.53)	30.71 (± 6.61)	30.70 (± 5.31)	26.23 (± 6.47)

Data shows the mean scores and standard deviations of the questionnaires that were filled out the evening before the experimental sessions

### Results of Self Report Anxiety Measured by STAI-State

The results of the repeated measure ANOVA on self-report state anxiety (STAI-S) with the within subject factors of time (Pre and Post intervention) and the between subject effects of Condition (Control, Biofeedback dominant fingers, Biofeedback nondominant fingers, Biofeedback dominant wrist, Biofeedback nondominant wrist) yielded a main effect of time *F* (1, 105) = 83.82, *p* < 0.001, η_p_^2^ = 0.44. There were no other significant main or interaction effects. See Table 2 for descriptive statistics and the results of the paired sample t-tests comparing pre and post intervention self-report anxiety.

**Table 2:** Pre and post intervention STAI state scores

Condition		Pre session		Post session		*t*	*p*	Cohen’s d
		Mean	SD	Mean	SD			
Control		40.56	8.93	35.89	6.84	3.36	0.004	0.79
Biofeedback	Nondominant fingers	37.09	7.78	33.04	4.49	3.17	0.004	0.66
	Nondominant wrists	37.86	10.56	31.18	7.05	6.09	< 0.001	1.30
	Dominant fingers	40.75	8.14	34.20	7.59	3.33	0.003	0.68
	Dominant wrists	40.83	9.38	32.91	6.49	6.48	< 0.001	1.35

### Results of Skin Conductance Level

The results of the repeated measure ANOVA on arousal measured by skin conductance level with the within subject factors of Time (Pre and Post intervention) and Measurement site (Intervention site and Reference site) and the between subject effects of Condition (Control, Biofeedback dominant fingers, Biofeedback nondominant fingers, Biofeedback dominant wrist, Biofeedback non-dominant wrist) yielded a main effect of Time, *F* (1, 72) = 58.36, *p* < 0.001, *η*_*p*_^*2*^ = 0.45, and a main effect of Measurement Site, *F* (1, 72) = 20.18, *p* < 0.001, *η*_*p*_^*2*^ = 0.22. The analysis also resulted in an interaction effect of Time and Condition, *F* (4, 72) = 3.02, *p* = 0.023, *η*_*p*_^*2*^ = 0.14 and Measurement site and Condition, *F* (4, 72) = 4.93, *p* = 0.001, *η*_*p*_^*2*^ = 0.22. The analysis yielded a three-way interaction effect of Time, Measurement Site, and Condition, *F* (4, 72) = 3.03, *p* = 0.023, *η*_*p*_^*2*^ = 0.14. See Table 3 for descriptive statistics and the results of the paired sample t tests.

**Table 3:** Average SCL pre and post intervention

Condition			SCL firs 30 s		SCL last 30 s		*t*	*p*	Cohen’s d
			Mean	SD	Mean	SD			
Control (N = 12)		Nondominant fingers	6.85	3.01	6.45	4.09	0.45	0.66	0.13
		Nondominant wrist	3.06	3.03	2.76	4.39	0.49	0.64	0.14
Biofeedback	Nondominant fingers (*N* = 16)	Nondominant fingers	5.88	2.74	3.84	2.39	3.25	0.005	0.81
		Dominant fingers	5.54	2.77	3.73	2.52	2.77	0.014	0.69
	Nondominant wrist (*N* = 15)	Nondominant wrist	3.55	3.07	1.59	1.63	2.97	0.01	0.77
		Nondominant fingers	7.18	3.39	3.03	2.65	6.04	< 0.001	1.56
	Dominant fingers (*N* = 17)	Dominant fingers	5.60	3.80	3.75	3.94	5.54	< 0.001	1.35
		Nondominant fingers	5.48	4.04	3.94	4.00	4.07	< 0.001	0.99
	Dominant wrists (*N* = 17)	Dominant wrists	4.50	3.23	2.36	2.74	3.67	0.002	0.89
		Nondominant fingers	5.46	2.64	3.89	2.82	2.38	0.03	0.58

T and p values and effect sizes (Cohen’s d) are based on the paired sample t tests comparing first and last 30 s averages

### Results of Non-specific Responses

The results of the repeated measure ANOVA on non-specific responses with the within subject factors of Time (Pre and Post intervention) and Measurement site (Intervention and Reference site) and the between subject effects of Condition (Control, Biofeedback dominant fingers, Biofeedback nondominant fingers, Biofeedback dominant wrist, Biofeedback nondominant wrist) yielded a main effect of time *F* (1, 72) = 12.26, *p* < 0.001, *η*_*p*_^*2*^ = 0.15, and a main effect of measurement site *F* (1, 72) = 17.90, *p* < 0.001, *η*_*p*_^*2*^ = 0.20. The analysis also showed a significant interaction effect of Condition and Measurement site *F* (1, 72) = 3.52, *p* = 0.011, *η*_*p*_^*2*^ = 0.16. There were no other significant effects. There were fewer non-specific responses in the beginning of the session compared to the end of the session, and there were more non-specific responses measured from the reference site compared to the intervention sites. See Table 4 for descriptive statistics and for the results of the paired sample t tests.

**Table 4:** Number of SCRs pre and post intervention

Condition			nSCR firs 30 s		nSCR last 30 s		*t*	*p*	Cohen’s d
			Mean	SD	Mean	SD			
Control (*N* = 12)		Nondominant fingers	14.83	4.02	11.58	5.09	3.30	0.007	0.95
		Nondominant wrist	7.67	6.51	4.50	6.43	1.69	0.120	0.31
Biofeedback	Nondominant fingers (*N* = 16)	Nondominant fingers	12.81	6.74	13.19	8.69	−0.19	0.849	−0.05
		Dominant fingers	12.75	5.37	12.06	7.24	0.42	0.679	0.11
	Nondominant wrist (*N* = 15)	Nondominant wrist	9.20	7.14	4.13	8.27	2.06	0.058	0.53
		Nondominant fingers	14.33	6.84	8.87	6.29	2.47	0.027	0.64
	Dominant Fingers (*N* = 17)	Dominant fingers	13.24	5.18	10.12	8.00	1.60	0.130	0.39
		Nondominant fingers	14.47	5.38	9.59	6.87	2.17	0.046	0.53
	Dominant wrists (*N* = 17)	Dominant wrists	9.65	6.07	9.06	12.43	0.22	0.827	0.05
		Nondominant fingers	14.06	6.09	12.94	6.27	0.46	0.652	0.11

The t and p values and the effect sizes (Cohen’s d) are based on the t tests comparing the number of SCRs

### Results of Response Amplitudes

The results of the repeated measure ANOVA on non-specific response amplitudes with the within subject factors of Time (Pre and Post intervention) and Measurement site (Intervention and Reference site) and the between subject effects of Condition (Control, Biofeedback dominant fingers, Biofeedback nondominant fingers, Biofeedback dominant wrist, Biofeedback nondominant wrist) yielded a main effect of time *F* (1, 72) = 4.54, *p* < 0.034, *η*_*p*_^*2*^ = 0.06, and a main effect of Measurement site *F* (1, 72) = 4.07, *p* < 0.047, *η*_*p*_^*2*^ = 0.05. The analysis also resulted in a significant interaction effect of Group and Measurement site *F* (1, 72) = 4.51, *p* = 0.003, *η*_*p*_^*2*^ = 0.20. There were no other significant effects. Overall, the amplitude of responses tended to be lower at the end of the session compared to the beginning of the session. Amplitudes measured were higher on the reference sites compared to the intervention sites. See Table 5 for descriptive statistics and the results of the paired sample t tests.

**Table 5:** Pre and Post session SCR amplitude averages

Condition			Amplitudes firs 30 s		Amplitudes last 30 s		*t*	*p*	Cohen’s d
			Mean	SD	Mean	SD			
Control (*N* = 12)		Nondominant fingers	3.79	2.95	4.37	4.23	−0.64	0.536	−0.18
		Nondominant wrist	0.75	1.11	1.11	2.70	−0.61	0.555	−0.18
Biofeedback	Nondominant fingers (*N* = 16)	Nondominant fingers	3.05	2.46	2.49	4.68	0.74	0.473	0.18
		Dominant fingers	2.73	2.00	2.47	4.85	0.27	0.79	0.07
	Nondominant wrist (*N* = 15)	Nondominant wrist	1.08	1.27	0.08	0.20	3.18	0.007	0.82
		Nondominant fingers	2.81	2.14	0.84	1.40	3.24	0.006	0.84
	Dominant Fingers (*N* = 17)	Dominant fingers	3.05	2.46	2.42	4.68	0.74	0.473	0.18
		Nondominant fingers	2.73	2.00	2.47	4.85	0.27	0.792	0.07
	Dominant wrists (*N* = 17)	Dominant wrists	1.51	1.57	0.73	1.34	2.59	0.020	0.63
		Nondominant fingers	2.50	3.35	2.36	2.74	0.125	0.902	0.03

The *t* and *p* values and the effect sizes (Cohen’s d) are based on the t tests comparing the amplitudes of SCRs

### Correlation Between Self−report Anxiety and Physiological Measures

Spearman correlation was conducted to analyze the association between STAI-state pre intervention, STAI-state post intervention, change in STAI-state and average SCL in the first 30 s, average SCL in the last 30 s, change in SCL, average number of SCRs in the first 30 s, average number of SCRs in the last 30 s, change in the number of SCRs between the first and last 30 s, average SCR amplitude in the first 30 s, average SCR amplitude in the last 30 s, change in SCR amplitude between the first and last 30 s. After adjusting for multiple comparisons (Bonferoni adjustments) none of the correlations remained significant.

## Study 2

### Method

A follow-up study was carried out to validate the main findings of the initial study, adhering to the same ethical standards and recruitment strategies. This second study involved 39 university students—including 29 females—who participated for course credit. The participants had an average age of 21.8 years with a standard deviation of 3.2. Employing the same methodology and procedures, the second study was conducted at the identical location as the first. The primary distinction between the two studies was the configuration of the treatment groups. In the follow-up study, participants were assigned to two specific treatment groups—one receiving biofeedback from the nondominant fingers and the other from the nondominant wrists—while maintaining the same control condition as in the first study. Data handling and Statistical analysis for both self-report and EDA data followed what was outlined in the first study.

### Results

One-way ANOVA was conducted to reveal any pretreatment differences in Anxiety, Stress, and Depression among conditions. The analysis did not yield significant differences among the conditions (See Table 6 for details).

**Table 6:** Descriptive statistics of the Battery of questionnaire

Self report measures	Control group *N* = 14	Nondominant fingers *N* = 12	Nondominant wrist *N* = 13
Stai Trait	51.28 (± 8.70)	51.25 (± 11.27)	52.69 (± 7.93)
Dass Stress	8.35 (± 4.29)	8.50 (± 4.81)	8.53 (± 4.17)
Dass Anxiety	4.64 (± 4.03)	3.92 (± 2.87)	4.53 (± 3.66)
Dass Depression	5.57 (± 5.03)	5.33 (± 4.14)	5.92 (± 4.07)
Perceived Stress Scale (PSS)	22.35 (± 3.56)	21.33 (± 3.23)	22.61 (± 3.28)

Data shows the mean scores and standard deviations of the questionnaires that were filled out the evening before the experimental sessions

#### Results of Self Report Anxiety Measured by STAI-State

The results of the repeated measure ANOVA on self-report state anxiety (STAI-S) with the within subject factors of Time (Pre and Post intervention) and the between subject effects of Condition (Control, Biofeedback non-dominant fingers, Biofeedback non-dominant wrist) yielded a main effect of time *F* (1, 36) = 17.79, *p* < 0.001, *η*_*p*_^*2*^ = 0.33. There were no other significant effects. Overall, all groups experienced a reduction in their anxiety (Table 7).

**Table 7:** STAI-state scores pre and post intervention

Condition		Pre session		Post session		*t*	*p*	Cohen’s d
		Mean	SD	Mean	SD			
Control (N = 14)		40.21	13.15	36.36	9.30	1.97	0.07	0.53
Biofeedback	Nondominant fingers (*N* = 12)	35.33	7.62	32.33	5.26	1.96	0.075	0.57
	Nondominant wrists (*N* = 13)	40.23	7.47	34.92	5.72	3.93	0.002	1.09

#### Results of Skin Conductance Level

The results of the repeated measure ANOVA on arousal measured by skin conductance level with the within subject factors of Time (Pre and Post intervention) and Measurement site (Intervention site and Reference site) and the between subject effects of Condition (Control, Biofeedback nondominant fingers, Biofeedback nondominant wrist) yielded a main effect of Time *F* (1, 21) = 20.79, *p* < 0.001, *η*_*p*_^*2*^ = 0.50. The analysis also resulted in an interaction effect of Measurement site and Condition *F* (2, 21) = 5.06, *p* = 0.016, *η*_*p*_^*2*^ = 0.32. A marginally significant three-way interaction effect of Time, Measurement Site and Condition *F* (2, 21) = 3.01, *p* = 0.071, *η*_*p*_^*2*^ = 0.22 was found. No other significant effects were observed (Table 8). The analysis confirmed the results of the first experiment.

**Table 8:** SCL averages pre and post intervention

Condition			SCL first 30 s		SCL last 30 s		*t*	*p*	Cohen’s d
			Mean	SD	Mean	SD			
Control (*N* = 9)		Nondominant fingers	9.65	4.22	6.69	4.62	2.54	0.035	0.85
		Nondominant wrist	4.70	3.61	4.08	5.89	0.60	0.563	0.15
Biofeedback	Nondominant fingers (*N* = 5)	Nondominant fingers	7.29	1.96	6.04	4.26	0.76	0.488	0.45
		Dominant fingers	7.69	4.31	3.39	2.69	2.24	0.088	1.00
	Nondominant wrist (*N* = 10)	Nondominant wrist	4.20	2.05	2.26	2.21	2.81	0.020	0.89
		Nondominant fingers	13.10	13.13	9.26	10.13	3.01	0.015	0.95

The t and p values and the effect sizes (Cohen’s d) are based on the t tests comparing SCLs

#### Results of the Number of Non-specific Responses

The results of the repeated measure ANOVA on non-specific responses with the within subject factors of Time (Pre and Post intervention) and Measurement site (Intervention and Reference site) and the between subject effects of Condition (Control, Biofeedback nondominant fingers, Biofeedback nondominant wrist) yielded a main effect of time *F* (1, 21) = 21.34, *p* < 0.001, *η*_*p*_^*2*^ = 0.50. The analysis also showed a significant interaction effect of Condition and Measurement site *F* (2, 21) = 10.40, *p* = 0.001, *η*_*p*_^*2*^ = 0.50. Also, a marginally significant three-way interaction effect of Time, Measurement Site and Condition *F* (2, 21) = 3.18, *p* = 0.062, *η*_*p*_^*2*^ = 0.23 was found. There were no other significant effects. There were fewer non-specific responses in the beginning of the session compared to the end of the session, and there were more non-specific responses measured from the reference site compared to the intervention sites (Table 9).

**Table 9:** Number of SCRs pre and post intervention

Condition			nSCR first 30 s		nSCR last 30 s		*t*	*p*	Cohen’s d
			Mean	SD	Mean	SD			
Control (N = 9)		Nondominant fingers	15.11	4.31	13.56	3.47	0.99	0.352	0.33
		Nondominant wrist	4.70	4.94	4.08	6.26	2.26	0.053	0.76
Biofeedback	Nondominant fingers (*N* = 5)	Nondominant fingers	11.89	5.45	6.78	8.04	1.37	0.243	0.61
		Dominant fingers	13.60	3.21	5.40	6.54	2.07	0.107	0.93
	Nondominant wrist (*N* = 10)	Nondominant wrist	13.6	8.62	3.5	5.86	3.43	0.008	1.08
		Nondominant fingers	17.7	3.86	13.9	7.20	1.97	0.081	0.62

The t and p values and the effect sizes (Cohen’s d) are based on the t tests comparing SCLs

#### Results of Response Amplitudes

The results of the repeated measure ANOVA on non-specific response amplitudes with the within subject factors of Time (Pre and Post intervention) and Measurement site (Intervention and Reference site) and the between subject effects of Condition (Control, Biofeedback from nondominant fingers, Biofeedback nondominant wrist) yielded an interaction effect of Condition and Measurement site *F* (2, 21) = 4.50, *p* = 0.024, *η*_*p*_^*2*^ = 0.30. There were no other significant effects. Amplitudes measured were higher on the reference site compared to the intervention site (Table 10).

**Table 10:** SCR amplitudes pre and post intervention

Condition			Amplitudes first 30 s		Amplitudes last 30 s		*t*	*p*	Cohen’s d
			Mean	SD	Mean	SD			
Control (*N* = 9)		Nondominant fingers	4.10	2.67	6.03	9.14	−0.66	0.531	−0.22
		Nondominant wrist	1.15	1.16	1.22	2.56	−0.13	0.902	−0.04
Biofeedback	Nondominant fingers (*N* = 5)	Nondominant fingers	6.26	4.56	5.27	5.81	0.24	0.820	0.10
		Dominant fingers	1.11	0.95	0.37	0.66	1.56	0.195	0.70
	Nondominant wrist (*N* = 10)	Nondominant wrist	0.82	0.61	0.41	1.13	1.21	0.257	0.38
		Nondominant fingers	10.86	14.68	7.13	14.47	4.72	0.001	1.49

The *t *and *p* values and the effect sizes (Cohen’s d) are based on the *t* tests comparing SCR amplitudes

#### Correlation Between Self Report Anxiety and Physiological Measures

Spearman correlation was conducted to analyze the association between variables detailed in the result section of study 1. The analysis revealed no significant correlations, replicating the results of study 1.

## Discussion

The present study aimed to evaluate the efficacy of a one-time electrodermal biofeedback in reducing anxiety, specifically exploring the potential of the wrist as a biofeedback site. The study also explored the relationship between self-report anxiety and EDA measured during biofeedback. Results signal preliminary efficacy of EDA biofeedback in reducing self-reported anxiety and SCL, SCR and non-specific responses. The study found that the efficacy of EDA biofeedback measured from the wrists is not significantly different from EDA biofeedback measured from the fingers. Furthermore, the results indicate no linear relationship between self-report anxiety and EDA measured during biofeedback.

### Self-report Anxiety

Our results showed that self-report anxiety was reduced in all conditions. These findings compare very well with studies reporting reductions in anxiety following EDA biofeedback sessions (Biswas et al., 1995; Dillon et al., 2016; Janka et al., 2017). The results are also in line with results from other studies that reported control conditions that were as effective in reducing anxiety as biofeedback. For example, Weerdmeester et al. (2020) reported that a deep breathing control condition was just as effective in reducing anxiety as virtual reality-based biofeedback. Prinsloo et al. (2013) found that after a single session of HRV biofeedback, the control group–watching pre-recorded feedback—experienced significant anxiety reduction. This implies a strong impact of a controlled environment and the act of focused engagement in reducing anxiety (Zhang et al., 2023). Nevertheless, we believe there is a distinct benefit in learning to actively control arousal levels. In real-life situations, the ability to willingly reduce physiological arousal can be highly advantageous, as it is not always possible to sit down in a controlled environment. Being able to manage arousal voluntarily enables individuals to handle stress more effectively in various settings, enhancing their overall resilience and mental health. Importantly, feedback obtained from the wrist had no significantly different effects on self-reported anxiety compared to the traditional feedback sites, this result was also supported by the follow up study. This contributes to the potential of wrist-administered biofeedback as a viable method for anxiety management. It also provides evidence that wearable devices, which are often placed on the wrists, are a feasible option for providing feedback.

However, it is important to note that in both experiments, our sample did not exhibit high levels of state anxiety, and their trait anxiety was only moderately elevated. This suggests that the outcomes might differ if participants were introduced to a stressor that significantly increases their state anxiety. Future experiments should consider exposing participants to a stressor to better generalize the findings to real-world stressful situations, as well as conducting studies in a clinical sample.

### Physiological Arousal

Our findings indicate that SCL decreased across participants, a trend predominantly driven by the intervention groups. In the intervention groups there is a significant decrease between the beginning and the end of the sessions compared to the control group where we observed no significant decrease. This observation underscores the efficacy of electrodermal biofeedback in reducing physiological arousal, which is crucial given that heightened and prolonged physiological arousal contributes to stress-related diseases, including cardiovascular issues and immune system disorders. However, these results were not consistently replicated in the follow-up study, in which in the control condition participants also exhibited some SCL reduction, and the group receiving feedback from the fingers did not show significant reduction in SCL. Although, we had low sample size in the biofeedback from the fingers condition in the second study due to loss of data. We also, as expected, measured lower skin conductance levels from the wrist compared to the fingers (Kasos et al., 2020b). This pattern was evident in our data and explains the group interaction effect observed: The reference device, consistently positioned on the nondominant hand’s fingers, registered higher skin conductance levels in scenarios where feedback was derived from the wrists. Additionally, a noteworthy three-way interaction effect emerged, particularly for the group receiving feedback from the nondominant wrist, the reference device on the nondominant fingers showed a steeper decrease in skin conductance levels compared to other groups. This finding, which was partially supported by the follow-up study, is intriguing and merits further future investigation.

Our results suggest that the number of non-specific responses on average decreased between the start and end of the biofeedback session. This result is in line with studies showing lower number of non- specific responses at the end of relaxation sessions (Kasos et al., 2020a, b, c). As expected, the number of responses measured from the wrists was lower than those measured from the fingers. The amplitude of non-specific responses exhibited smaller values at the end of the session compared to the beginning. This finding aligns with results showing reduced amplitude responses following relaxation inductions (Kasos et al., 2020a, b, c). Furthermore, in our experience there is a high positive correlation between response amplitudes and SCL, which may also contribute to this observation. The main effect of the measurement site is not surprising, given previous results that show lower amplitude responses recorded from the wrists compared to the fingers (Kasos et al., 2020a). The interaction effect of measurement site and condition was replicated in the second experiment, which suggests, that different conditions influence response amplitudes differently. Deciphering whether these differences impact anxiety reduction will require future studies.

Parnandi et al. (2017) compared HRV and EDA biofeedback and suggested that EDA biofeedback was not as effective compared to HRV and breathing biofeedback because EDA is low on voluntary control, but has high arousal selectivity, therefore it is more difficult to gain control over EDA compared to the other biofeedback methods. We, on the other hand, demonstrated in these experiments that most participants (over 82%) were able to gain control over their EDA in a single, short 10-min session. In the first experiment participants’ mean SCL levels were 3.14 (SD = 2.39, Min = 0.09, Max = 5.88) in the second experiment their mean SCL levels were 5.75 (SD = 3.16, Min = 0.13, Max = 10.06). These low levels may make it difficult to lower SCL since there is an increased floor effect. Indeed, it is our experience that most people are able to gain control over their sympathetic responses. Thus, the underperformance of EDA biofeedback compared to other methods may result from other factors. It may well be that parasympathetic involvement plays a significant role in anxiety reduction and targeting only the sympathetic nervous system is not as sufficient. Unfortunately, answering this question is not in the scope of this study, but future research should investigate this further.

Our results also resonate well with new theories in EDA, such as Rosalind Picard's Multiple Arousal Theory (MAT; Picard et al., 2015). MAT posits that arousal is not a uniform phenomenon across the body but is instead driven by different central mechanisms depending on the site of measurement. This theory challenges the traditional view that EDA is a singular, uniform indicator of physiological arousal. According to the theory, if arousal were uniform across the body, there would be no deviations between biofeedback and reference sites. However, the observed deviations suggest that arousal can be influenced on a dermatome and this influence does not translate uniformly to other dermatomes. These deviations are observable in SCL, SCR amplitude, and the number of non-specific responses. Moreover, after exploring the data in the biofeedback groups the correlation between measurement sites significantly decreased from the beginning to the end of the session, and this was not observed in the control condition. This finding also supports MAT, as EDA can be influenced site-specifically.

### Association Between Self Report Anxiety and EDA

Our study found no significant correlation between EDA components and self-reported anxiety. When one performs electrodermal biofeedback for anxiety or stress reduction, they aim to lower sympathetic arousal in the hope of also reducing self-reported anxiety. Therefore, we would expect a clear association between the two, making the lack of correlation particularly intriguing. For example, Nagai et al. (2019) when examining EDA biofeedback effects of epileptic seizures, found that EDA amplitudes were associated with epileptic seizure frequency. Another study also found significant correlation between the change in EDA during biofeedback and seizure frequency (Schoenberg et al., 2012). Similarly in EDA biofeedback in anxiety reduction some association between measures would be desirable. Supporting our results, research by Strohmaier et al. (2020) did not establish a clear link between self-reported anxiety and EDA levels. Indeed, in our experiment some participants reported lower anxiety by the end of the session, despite an increase in their SCL. This finding is in line with the ones of Schoenberg et al. (2012) who reported that participants who were trained to increase their EDA during feedback did not show elevated self-report anxiety.

Given these findings, the complex relationship between autonomic measures and self-reported anxiety may suggest that reducing EDA alone might not directly translate to lower levels of anxiety. It is possible that focusing on a task during biofeedback sessions helps distract participants from anxious thoughts, contributing to a reduction in anxiety despite physiological measures indicating otherwise. This is supported by cases where participants reported lower anxiety even when their sympathetic arousal increased by the end of the session. Additionally, in our study participants were aware of the main goal of the study (anxiety reduction), it might have influenced participants self-report introducing a bias or a strong suggestion that their anxiety would be improved by biofeedback.

While the lack of a straightforward correlation between EDA and self-reported anxiety may seem counterintuitive, it does not undermine the value of biofeedback. Biofeedback offers a unique training tool for self-regulation and complements other anxiety management techniques. Integrating both autonomic indicators and self-reported measures provides a comprehensive understanding of anxiety and treatment responses, helping clinicians develop more effective, holistic interventions. Future studies should further explore these dynamics to clarify the relationship and enhance biofeedback's efficacy in anxiety management.

## Conclusion

In conclusion, our results demonstrate evidence of the feasibility of EDA biofeedback in reducing state anxiety. Its efficacy was demonstrated in lowering self-reported anxiety and SCL, SCR and non-specific responses. Feedback from the wrist—a common placement for self-monitoring devices and wearable technology—was found to be not significantly different in reducing state anxiety from traditional placement sites. Our results emphasize the importance of recognizing the complexity and site-specific nature of electrodermal responses. By acknowledging that different parts of the body may have distinct central drivers of arousal, researchers can develop more accurate and effective biofeedback interventions in the future.

Our study faces limitations due to the loss of data and a sample predominantly comprised of university students who participated in exchange for course credit. Additionally, the biofeedback intervention was confined to a single session lasting only ten minutes. It would be interesting to see if longer (20 or 30 min) single biofeedback sessions had more efficacy compared to a 10-min session used in these studies. We also acknowledge that a single session biofeedback may not be as effective as multiple sessions. Future research should investigate the long-term efficacy of EDA biofeedback to better understand its longitudinal impact. Future studies ought to look into the dynamics of how changes in the ability to control and influence EDA correlate with variations in self-reported anxiety. These investigations will provide deeper insights into the psychological mechanisms underpinning biofeedback and its potential in anxiety management. Furthermore, we examined a non-clinical sample which generally has relatively low levels of anxiety, creating a floor effect that may bias the results towards lower overall reductions in both EDA and self-report anxiety. Accordingly, a future study with a similar design could be conducted on clinical population. Moreover, in the experimental design the control condition was reading in a quite room, which could be considered relaxing in itself. Control conditions in biofeedback studies many times include reading or breathing exercises and, in some cases, sham biofeedback. Finding an appropriate control for biofeedback studies is challenging, since conditions which are arousing could artificially inflate the efficacy of biofeedback. On the other hand, conditions which provide excessive relaxation may result in deflating the effect of biofeedback. Perhaps future studies could look into using more than one type of control condition. A potential further limitation of our study is the lack of credibility ratings, which makes it difficult to determine whether the observed anxiety reduction was influenced by differences in participants' belief in the intervention.

## Data Availability

Data that support the findings are available Uppon request.

## References

[CR1] Attwood, A. S., Easey, K. E., Dalili, M. N., Skinner, A. L., Woods, A., Crick, L., Ilett, E., Penton-Voak, I. S., & Munafò, M. R. (2017). State anxiety and emotional face recognition in healthy volunteers. *Royal Society Open Science,**4*(5), 160855. 10.1098/rsos.160855 28572987 10.1098/rsos.160855 PMC5451788

[CR2] Bandelow, B., & Michaelis, S. (2015). Epidemiology of anxiety disorders in the 21st century. *Dialogues in Clinical Neuroscience,**17*(3), 327. 10.31887/dcns.2015.17.3/bbandelow 26487813 10.31887/DCNS.2015.17.3/bbandelow PMC4610617

[CR3] Bandelow, B., Michaelis, S., & Wedekind, D. (2017). Treatment of anxiety disorders. *Dialogues in Clinical Neuroscience,**19*(2), 93–106. 10.4324/9780203728215-32 28867934 10.31887/DCNS.2017.19.2/bbandelow PMC5573566

[CR4] Benedek, M., & Kaernbach, C. (2010a). A continuous measure of phasic electrodermal activity. *Journal of Neuroscience Methods,**190*(1), 80–91. 10.1016/j.jneumeth.2010.04.028 20451556 10.1016/j.jneumeth.2010.04.028 PMC2892750

[CR5] Benedek, M., & Kaernbach, C. (2010b). Decomposition of skin conductance data by means of nonnegative deconvolution. *Psychophysiology,**47*(4), 647–658. 10.1111/j.1469-8986.2009.00972.x 20230512 10.1111/j.1469-8986.2009.00972.x PMC2904901

[CR6] Bhambri, E. (2021). Effect of biofeedback assisted psychological interventions of anxious sports persons. *International Journal of Physical Education, Sports and Health,**8*(5), 246–250.

[CR7] Biswas, A., Biswas, D., & Chattopadhyay, P. K. (1995). Cognitive behaviour therapy in generalised anxiety disorder. *Indian Journal of Clinical Psychology*, *22*.

[CR8] Boucsein, W. (2012). *Electrodermal activity* (*2*^*nd*^* edition*). Springer. 10.1007/978-1-4614-1126-0

[CR9] Cohen, S., Kamarck, T., & Mermelstein, R. (1983). A global measure of perceived stress. *Journal of Health and Social Behavior.,**24*(4), 385. 6668417

[CR10] Craske, M. G., Hermans, D., & Vervliet, B. (2018). State-of-the-art and future directions for extinction as a translational model for fear and anxiety. *Philosophical Transactions of the Royal Society B: Biological Sciences,**373*(1742), 20170025. 10.1098/rstb.2017.0025 10.1098/rstb.2017.0025 PMC5790824 29352025

[CR11] Craske, M. G., Treanor, M., Conway, C. C., Zbozinek, T., & Vervliet, B. (2014). Maximizing exposure therapy: An inhibitory learning approach. *Behaviour Research and Therapy,**58*, 10–23. 10.1016/j.brat.2014.04.006 24864005 10.1016/j.brat.2014.04.006 PMC4114726

[CR12] Dawson, M. E., Schell, A. M., & Filion, D. L. (2007). *The electrodermal system*. Cambridge University Press. 10.1016/j.ces.2007.04.037

[CR13] Dillon, A., Kelly, M., Robertson, I. H., & Robertson, D. A. (2016). Smartphone applications utilizing biofeedback can aid stress reduction. *Frontiers in Psychology,**7*, 1–7. 10.3389/fpsyg.2016.00832 27378963 10.3389/fpsyg.2016.00832 PMC4911859

[CR14] Gustavsson, A., Svensson, M., Jacobi, F., Allgulander, C., Alonso, J., Beghi, E., Dodel, R., Ekman, M., Faravelli, C., Fratiglioni, L., Gannon, B., Jones, D. H., Jennum, P., Jordanova, A., Jönsson, L., Karampampa, K., Knapp, M., Kobelt, G., Kurth, T., & Olesen, J. (2011). Cost of disorders of the brain in Europe 2010. *European Neuropsychopharmacology,**21*(10), 718–779. 10.1016/j.euroneuro.2011.08.008 21924589 10.1016/j.euroneuro.2011.08.008

[CR15] Hellhammer, D. H., Stone, A. A., Hellhammer, J., & Broderick, J. (2010). Measuring stress. *Encyclopedia of Behavioral Neuroscience* (pp. 186–191). Elsevier. 10.1016/B978-0-08-045396-5.00188-3

[CR16] Henry, J. D., & Crawford, J. R. (2005). The short-form version of the depression anxiety stress scales (DASS-21): Construct validity and normative data in a large non-clinical sample. *British Journal of Clinical Psychology,**44*(2), 29657. 10.1348/014466505X29657 10.1348/014466505X29657 16004657

[CR17] Janka, A., Adler, C., Brunner, B., Oppenrieder, S., & Duschek, S. (2017). Biofeedback training in crisis managers: A randomized controlled trial. *Applied Psychophysiology Biofeedback,**42*(2), 117–125. 10.1007/s10484-017-9360-6 28349228 10.1007/s10484-017-9360-6

[CR18] Kasos, K., Csirmaz, L., Vikor, F., Zimonyi, S., Varga, K., & Szekely, A. (2020). Electrodermal correlates of hypnosis: Current developments. *OBM Integrative and Complimentary Medicine,**5*(2), 1–20. 10.21926/obm.icm.2002017

[CR19] Kasos, K., Kekecs, Z., Csirmaz, L., Zimonyi, S., Vikor, F., Kasos, E., Veres, A., Kotyuk, E., & Szekely, A. (2020). Bilateral comparison of traditional and alternate electrodermal measurement sites. *Psychophysiology,**57*(11), 13645. 10.1111/psyp.13645 10.1111/psyp.13645 32931044

[CR20] Kasos, K., Kekecs, Z., Csirmaz, L., Zimonyi, S., Vikor, F., Kasos, E., Veres, A., Kotyuk, E., & Szekely, A. (2020). Bilateral comparison of traditional and alternate electrodermal measurement sites. *Psychophysiology,**57*, 11. 10.1111/psyp.13645 10.1111/psyp.13645 32931044

[CR21] Kasos, K., Zimonyi, S., Gönye, B., Koteles, F., Kotyuk, E., Varga, K., Veres, A., Szekely, A., Gonye, B., Köteles, F., Kasos, E., Kotyuk, E., Varga, K., Veres, A., & Szekely, A. (2019). Obimon: An open-source device enabling group measurement of electrodermal activity. *Psychophysiology,**56*, 1–15. 10.1111/psyp.13374 10.1111/psyp.13374 30950524

[CR22] Khanna, A., Paul, M., & Sandhu, J. S. (2007). Efficacy of two relaxation techniques in reducing pulse rate among highly stressed females. *Calicut Medical Journal,**5*(5), 3.

[CR23] Kilpatrick, D. G., Sutker, P. B., Roitzsh, J. C., & Mason, R. L. (2006). Self-reported fears and electrodermal responsiveness of high and low trait anxious subjects to fear of failure and other stressors. *Social Behavior and Personality: An International Journal,**3*(2), 205. 10.2224/sbp.1975.3.2.205

[CR24] Lau, J. Y. F., Eley, T. C., & Stevenson, J. (2006). Examining the state-trait anxiety relationship: A behavioural genetic approach. *Journal of Abnormal Child Psychology,**34*(1), 18. 10.1007/s10802-005-9006-7 10.1007/s10802-005-9006-7 16557359

[CR25] Leleu, V., Douilliez, C., & Rusinek, S. (2014). Difficulty in disengaging attention from threatening facial expressions in anxiety: A new approach in terms of benefits. *Journal of Behavior Therapy and Experimental Psychiatry,**45*(1), 203. 10.1016/j.jbtep.2013.10.007 24239586 10.1016/j.jbtep.2013.10.007

[CR26] Lewis, A. (1970). The ambiguous word “anxiety.” *International Journal of Psychiatry,**9*, 62–79. 4921639

[CR27] MacLean, D., Roseway, A., & Czerwinski, M. (2013). *MoodWings.,**25*, 1–8. 10.1145/2504335.2504406

[CR28] Morina, N., Maier, T., Bryant, R., Knaevelsrud, C., Wittmann, L., Rufer, M., Schnyder, U., & Müller, J. (2012). Combining biofeedback and narrative exposure therapy for persistent pain and PTSD in refugees: A pilot study. *European Journal of Psychotraumatology,**3*, 17660. 10.3402/ejpt.v3i0.17660 10.3402/ejpt.v3i0.17660 PMC3402113 22893834

[CR29] Nagai, Y., Critchley, H. D., Featherstone, E., Trimble, M. R., & Dolan, R. J. (2004). Activity in ventromedial prefrontal cortex covaries with sympathetic skin conductance level: A physiological account of a “default mode” of brain function. *NeuroImage,**22*(1), 243–251. 10.1016/j.neuroimage.2004.01.019 15110014 10.1016/j.neuroimage.2004.01.019

[CR30] Nagai, Y., Jones, C. I., & Sen, A. (2019). Galvanic skin response (GSR)/Electrodermal/Skin conductance biofeedback on epilepsy: A systematic review and meta-analysis. *Frontiers in Neurology,**10*, 377. 10.3389/fneur.2019.00377 31068887 10.3389/fneur.2019.00377 PMC6491510

[CR31] Naveteur, J., Freixa, I., & Baque, E. (1987). Individual differences in electrodermal activity as a function of subjects’ anxiety. *Personality and Individual Differences,**8*(5), 615–626. 10.1016/0191-8869(87)90059-6

[CR32] Palekar, T. J., Mokashi, M. G., Anwer, S., Kakrani, A. L., Khandare, S. D., & Alghadir, A. H. (2015). Effect of galvanic skin resistance-aided biofeedback training in reducing the pulse rate, respiratory rate, and blood pressure due to perceived stress in physiotherapy students. *Turkiye Fiziksel Tip Ve Rehabilitasyon Dergisi,**61*(2), 116–119. 10.5152/tftrd.2015.97957

[CR34] Parnandi, A., & Gutierrez-Osuna, R. (2017). Physiological modalities for relaxation skill transfer in biofeedback games. *IEEE Journal of Biomedical and Health Informatics,**21*(2), 361–371. 10.1109/JBHI.2015.2511665 28055927 10.1109/JBHI.2015.2511665

[CR33] Parnandi, A. R. (2017). Physiological self regulation with biofeedback games. *ProQuest Dissertations and Theses*, *May*, 208. https://manchester.idm.oclc.org/login?url=https://search.proquest.com/docview/2199613046?accountid=12253%0Ahttp://man-fe.hosted.exlibrisgroup.com/openurl/44MAN/44MAN_services_page?genre=dissertations+%26+theses&atitle=&author=Parnandi%2C+Avinash+Rao&volum

[CR35] Picard, R. W., Fedor, S., & Ayzenberg, Y. (2015). Multiple arousal theory and daily-life electrodermal activity asymmetry. *Emotion Review,**8*(1974), 62–75. 10.1177/1754073914565517

[CR36] Pop-Jordanova, N., & Pop-Jordanov, J. (2002). Psychophysiological comorbidity and computerized biofeedback. *International Journal of Artificial Organs,**25*(5), 429–433. 10.1177/039139880202500513 12074341 10.1177/039139880202500513

[CR37] Pop-Jordanova, N., & Pop-Jordanov, J. (2020). Electrodermal activity and stress assessment. *Prilozi,**41*(2), 5–15. 10.2478/prilozi-2020-0028 33011695 10.2478/prilozi-2020-0028

[CR38] Prinsloo, G. E., Derman, W. E., Lambert, M. I., & Laurie Rauch, H. G. (2013). The effect of a single episode of short duration heart rate variability biofeedback on measures of anxiety and relaxation states. *International Journal of Stress Management,**20*(4), 391–411. 10.1037/a0034777

[CR39] Raaijmakers, S. F., Steel, F. W., De Goede, M., Van Wouwe, N. C., Van Erp, J. B. F., & Brouwer, A. M. (2013). Heart rate variability and skin conductance biofeedback: A triple-blind randomized controlled study. In: *Proceedings - 2013 humaine association conference on affective computing and intelligent interaction, ACII 2013*, 289–293. 10.1109/ACII.2013.54

[CR40] Rosebrock, L. E., Hoxha, D., Norris, C., Cacioppo, J. T., & Gollan, J. K. (2017). Skin conductance and subjective arousal in anxiety, depression, and comorbidity: Implications for affective reactivity. *Journal of Psychophysiology,**31*(4), 145. 10.1027/0269-8803/a000176

[CR41] Rusciano, A., Corradini, G., & Stoianov, I. (2017). Neuroplus biofeedback improves attention, resilience, and injury prevention in elite soccer players. *Psychophysiology,**54*(6), 916–926. 10.1111/psyp.12847 28220500 10.1111/psyp.12847

[CR42] Scheutz, F. (1986). Electrodermal activity, dental anxiety, and fear of dentistry in a group of parenteral drug addicts. *European Journal of Oral Sciences,**94*(3), 248. 10.1111/j.1600-0722.1986.tb01760.x 10.1111/j.1600-0722.1986.tb01760.x 3461545

[CR43] Schoenberg, P. L. A., Sierra, M., & David, A. S. (2012). Psychophysiological investigations in depersonalization disorder and effects of electrodermal biofeedback. *Journal of Trauma and Dissociation,**13*(3), 311–329. 10.1080/15299732.2011.606742 22545565 10.1080/15299732.2011.606742

[CR44] Spielberger, C. D. (1966). The effects of anxiety on complex learning and academic achievement. *Anxiety and Behavior,* 361–398. Elsevier. 10.1016/B978

[CR45] Spielberger, C., Gorsuch, R., & Lushene, R. (1970). *STAI manual for the state-trait anxiety inventory*. Lushene Consulting Psychologists Press.

[CR46] Steinberg, E. P., & Schwartz, G. E. (1976). Biofeedback and electrodermal self-regulation in psychopathy. *Journal of Abnormal Psychology,**85*(4), 408–415. 10.1037/0021-843X.85.4.408 956508 10.1037//0021-843x.85.4.408

[CR47] Strohmaier, A. R., Schiepe-Tiska, A., & Reiss, K. M. (2020). A comparison of self-reports and electrodermal activity as indicators of mathematics state anxiety. *Frontline Learning Research,**8*(1), 16. 10.14786/flr.v8i1.427

[CR48] Teufel, M., Stephan, K., Kowalski, A., Käsberger, S., Enck, P., Zipfel, S., & Giel, K. E. (2013). Impact of biofeedback on self-efficacy and stress reduction in obesity: A randomized controlled pilot study. *Applied Psychophysiology Biofeedback,**38*(3), 177–184. 10.1007/s10484-013-9223-8 23760668 10.1007/s10484-013-9223-8

[CR49] Tinello, D., Kliegel, M., & Zuber, S. (2022). Does heart rate variability biofeedback enhance executive functions across the lifespan? A systematic review. *Journal of Cognitive Enhancement,**6*(1), 126–142. 10.1007/s41465-021-00218-3 35299845 10.1007/s41465-021-00218-3 PMC8901517

[CR50] Weerdmeester, J., Van Rooij, M. M. J. M., Engels, R. C. M. E., & Granic, I. (2020). An integrative model for the effectiveness of biofeedback interventions for anxiety regulation: Viewpoint. *Journal of Medical Internet Research,**22*(7), e14958. 10.2196/14958 32706654 10.2196/14958 PMC7413290

[CR51] Yahav, R., & Cohen, M. (2008). Evaluation of a cognitive-behavioral intervention for adolescents. *International Journal of Stress Management,**15*(2), 173–188. 10.1037/1072-5245.15.2.173

[CR52] Zhang, Y., Feng, L., & Duan, W. (2023). The impact of forest therapy programs on stress reduction: a systematic review. *Forests,**14*(9), 1851. 10.3390/f14091851

